# Primary Extramammary Paget's Disease in a Male Patient Exhibiting Genital Involvement Mimicking Clinical and Histopathological Features of Bowen's Disease: A Case Report

**DOI:** 10.7759/cureus.78242

**Published:** 2025-01-30

**Authors:** Diego Emilio Gómez López, Alma J Chávez-Rojas, Enrique J Gómez Flores, Ivett Miranda, Circe Ancona Castro, Yeudiel Suro Santos

**Affiliations:** 1 Department of Dermatology, Instituto de Seguridad y Servicios Sociales de los Trabajadores del Estado (ISSSTE) Monterrey Regional Hospital, Monterrey, MEX; 2 Department of Internal Medicine, Universidad Juárez del Estado de Durango, Durango, MEX; 3 Department of Dermatology, Sierra Madre Hospital, Monterrey, MEX; 4 Department of Pathology, Autonomous University of Nuevo Leon, Monterrey, MEX; 5 Department of General Surgery, Instituto Mexicano del Seguro Social, Hospital General de Zona No. 33, Monterrey, MEX

**Keywords:** bowen's disease, extramammary paget's disease, histopathology, immunohistochemistry, malignant tumor, pathology, skin cancer, skin surgery

## Abstract

Extramammary Paget’s disease (EMPD) is a rare cutaneous neoplasm characterized by the proliferation of malignant glandular-origin cells within the epidermis, occurring in areas other than the mammary areola. It is more prevalent in women (65%) than men (14%). Diagnosis can be confused with other neoplastic, inflammatory, or infectious conditions, often leading to initial inappropriate treatment with antifungals or topical steroids. Bowen’s disease, an in situ squamous cell carcinoma, can mimic EMPD both clinically and histologically. However, their diagnostic and therapeutic approaches differ. We present the case of a male patient with a dermatosis in the genital area affecting the pubis, base of the penis, and scrotum, manifesting as an erythematous scaly plaque with poorly defined edges. The lesion had been previously treated as an inflammatory dermatosis. Initially, the condition was diagnosed as Bowen’s disease through punch biopsy, but a definitive diagnosis of EMPD was established via excisional biopsy and immunohistochemistry. The case was resolved through surgical interventions and imaging studies to rule out involvement in other areas. This report highlights the complexity of diagnosing EMPD, emphasizing the importance of a proper diagnostic and therapeutic approach supported by specialized tools and multidisciplinary management.

## Introduction

Extramammary Paget’s disease (EMPD) is an uncommon cutaneous neoplasm characterized by the proliferation of malignant glandular-origin cells within the epidermis, occurring in areas outside the mammary areola. It develops in the skin or its appendages, primarily in regions with abundant apocrine glands. The most commonly affected areas include the vulva, perianal region, scrotum, penis, and axillae [1-3].

EMPD accounts for 6.5% of all Paget’s disease diagnoses. The vulva is the most common site, representing 65% of EMPD cases, and the condition is, therefore, more prevalent in women. EMPD affecting the male genital apparatus represents 14% of cases and is associated with adjacent cancers (e.g., prostate, bladder, testis, urethra, or kidney) in 11% of cases [4-6]. Underreporting is thought to occur due to the condition's low incidence and the challenges in achieving an accurate diagnosis, as it can be difficult to distinguish from other similar conditions [6]. Differential diagnosis is extensive, often mistaken for chronic eczema or infectious dermatitis treated with topical steroids or antifungals, given their higher prevalence. EMPD may also clinically resemble squamous cell carcinoma or Bowen’s disease, necessitating histopathological evaluation and multiple immunohistochemical markers to establish a precise diagnosis [6]. In this case report, we present a case of EMPD with genital involvement in a male patient mimicking the clinical and histopathological characteristics of Bowen’s disease.

This article was previously presented as a poster at the XLI Annual Meeting of Latin American Dermatologists Congress held from May 8 to 11, 2024, in Lima, Peru.

## Case presentation

We present the case of a 78-year-old male patient with a medical history of hypothyroidism, type 2 diabetes mellitus, and long-standing hypertension. His oncological-surgical history includes a squamous cell carcinoma on the helix of the right ear and a basal cell carcinoma on the right cheek, both successfully treated with surgical intervention.

The patient reported the onset of a condition in 2022 characterized by an erythematous lesion in the pubic region extending to the scrotum, described as nonpruritic irritation. Initially, he was treated with a high-potency topical steroid for two months by a private physician without resolution.

**Figure 1:**
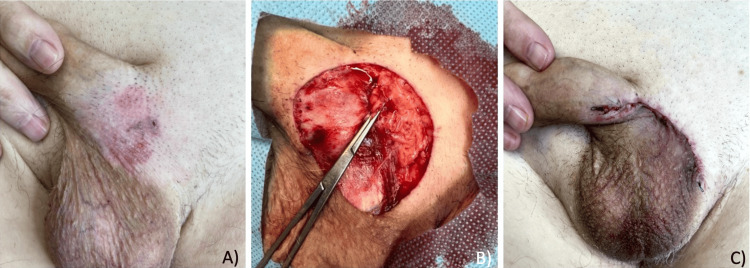
Clinical presentation and surgical management. (A) Erythematous scaly plaque measuring 4 cm × 2.2 cm in diameter with irregular, poorly defined borders. (B) Surgical resection of the lesion showing underlying tissue of the penile base, pubis, and scrotum. (C) Immediate result of the closure with intradermal suture pattern

Physical examination revealed a localized dermatosis in the pubis extending to the base of the penis and scrotum, characterized by a 4 cm × 2.2 cm oval erythematous scaly plaque with irregular, poorly defined edges (Figure 1A). Since the lesion did not resolve with the previous treatment with topical steroids, a punch biopsy was made, initially diagnosing Bowen’s disease. Subsequently, an excisional surgical resection was performed with a 1-cm margin (Figure 1B) and direct closure (Figure 1C).

**Figure 2:**
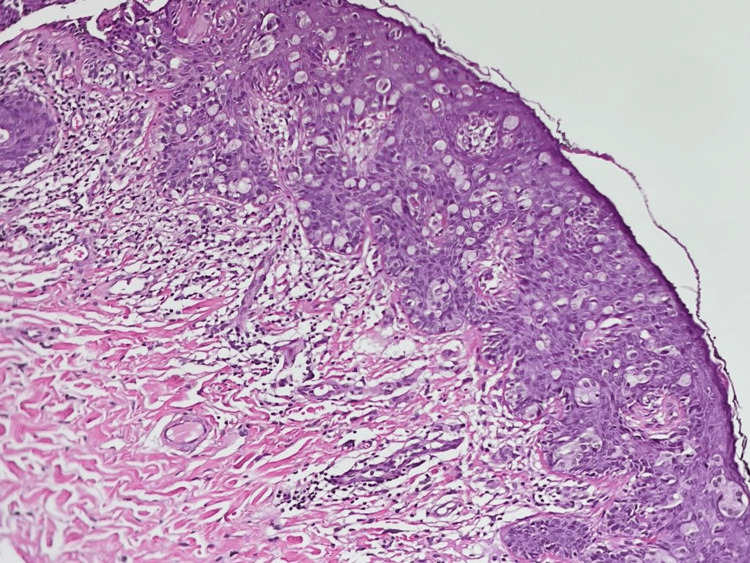
Skin biopsy with Hematoxylin and Eosin staining (10× magnification): tumor cells with abundant clear cytoplasm, pleomorphic nuclei, and prominent nucleoli are observed, arranged in small nests and isolated forms across all levels of the epidermis, exhibiting pagetoid migration

Histological sections revealed epidermal malignant epithelial neoplasia composed of round cells arranged in clusters and loosely distributed intraepithelial in a pagetoid pattern. These cells were large with abundant pale cytoplasm, nuclear atypia, and nucleoli, along with dermal invasion by individual cells and glandular structures (Figure 2).

**Figure 3:**
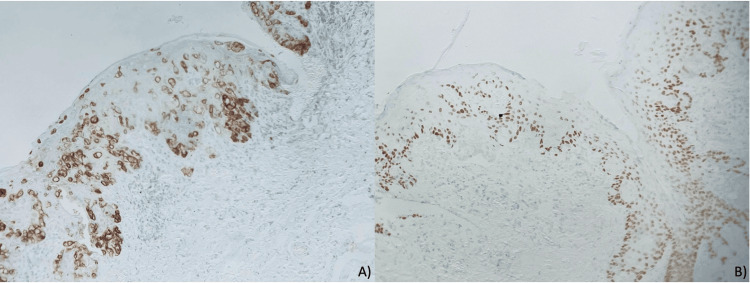
Skin biopsy with immunohistochemical staining (10× magnification). (A) Immunohistochemical staining for CEA shows positivity in neoplastic cells with pagetoid migration. (B) Immunohistochemical staining for P63 shows negativity in neoplastic cells and nuclear positivity in the squamous epithelial cells CEA: carcinoembryonic antigen; P63: tumor protein 63

Immunohistochemical staining showed positivity for carcinoembryonic antigen (CEA; see Figure 3A) and negativity for tumor protein 63 (P63; see Figure 3B) in neoplastic cells.

Despite two surgical interventions with positive margins, interdisciplinary management involving oncology and oncological surgery services enabled a third successful intervention with negative margins. Imaging studies, including computed tomography and positron emission tomography, revealed no genitourinary system involvement or metastatic dissemination. The patient is currently under close follow-up to monitor disease progression and remission.

## Discussion

EMPD is a rare condition with only a few reported cases. Its precise incidence is unknown, but it accounts for 6.5% of all cases of cutaneous Paget disease. It primarily affects patients between 50 and 80 years of age, with a peak incidence at 65.3 years. While it is more common among Caucasians and women, male predominance has been observed in Asia. EMPD in men is extremely rare and is often misdiagnosed as other skin neoplasms, reactive dermatitis, or infectious conditions. Identifying EMPD in its early stages is challenging due to its similarity to other entities. The diagnostic approach is frequently directed toward more common diseases, such as inflammatory dermatoses (chronic eczema, psoriasis, chronic lichen simplex, and lichen atrophicus) or infectious diseases, primarily fungal. It may also be confused with skin neoplasms such as squamous cell carcinoma, melanoma in situ, or Bowen's disease, which, in our case, was the initial diagnosis due to shared clinical characteristics and histopathological findings. This overlap highlights the importance of immunohistochemical markers, which are essential for distinguishing EMPD and achieving a definitive diagnosis [2,4-8].

It is common and reasonable for these lesions to initially raise suspicion for various diagnoses, such as contact dermatitis or fungal infections. However, when evaluating an unfavorable therapeutic response, a biopsy and application of immunohistochemical markers are necessary to rule out EMPD or other neoplasms, such as Bowen's disease, as in our case. Unfortunately, the correct diagnosis is often delayed by an average of two years, with reported delays extending up to 10 years or more [9].

In EMPD histopathology, a pagetoid pattern is observed in the epidermis. This pattern is characterized by large atypical cells with prominent nuclei and clear cytoplasm containing abundant mucin, which is sometimes eosinophilic [1,5]. Immunohistochemical markers include positive results for cytokeratin 7, gross cystic disease fluid protein-15 (GCDFP-15), and CEA and negative results for P63 and SOX10 [1]. The immunohistochemical marker SOX10 is important in the evaluation of an EMPD biopsy as it helps differentiate it from other neoplasms with similar histopathological features, particularly melanoma in situ or metastatic melanoma. SOX10 is a highly specific nuclear marker for neural crest-derived cells, including melanocytes and Schwann cells. EMPD does not express SOX10, whereas melanoma typically does [10].

While Bowen's disease often presents with more intense and diffuse erythema, EMPD is characterized by reddish milky areas indicative of localized increased vascular volume. This difference, although subtle, may aid in the differential diagnosis. Immunohistochemically, Bowen’s disease is positive for P63 and CEA. In our case, this diagnosis was ruled out due to a negative result for P63 [6,7,11].

EMPD can be classified as primary or secondary. Primary EMPD is associated with apocrine adenocarcinomas with intraepithelial involvement of the epidermis and may progress to dermal invasion, dissemination to lymphatic and blood vessels, and metastasis. Secondary EMPD, on the other hand, originates from an internal neoplasm near or distant to the lesion, spreading to the skin through tumor cells with epidermotropic behavior. The prognosis of the disease is good when diagnosed in early stages, achieving survival rates greater than 90% at five years in primary EMPD limited to the epidermis. However, secondary EMPD has a poor prognosis impacted by the underlying visceral malignancy. The mortality rate for secondary EMPD can be greater than 50%. Primary EMPD is more common in penoscrotal involvement. Markers cytokeratin 20 and GCDFP-15 are used to classify EMPD as primary or secondary. CEA is positive in nearly 100% of secondary EMPD cases, so a negative test for this antigen strongly suggests a primary form, ruling out the possibility of an adjacent tumor. However, screening for primary neoplasms is still necessary. Imaging studies are necessary to rule out the involvement of other systems, as in the case presented, yielding no abnormalities [1,4,6,7,11-13].

Differentiating between primary and secondary EMPD can sometimes be difficult, both clinically and histopathologically. This classification is important because each type has different treatment plans and prognoses, making appropriate diagnosis with a histopathological and immunohistochemical focus essential [7].

The average interval between EMPD diagnosis and the detection of an internal malignancy is five years. About 18% of adenocarcinomas, mainly of anorectal, breast, prostate, or urothelial origin, are diagnosed within one year of EMPD diagnosis [4].

The first-line treatment for EMPD is surgery, which involves wide local excision or Mohs micrographic surgery [1,5]. Wide local excision is considered the treatment of choice, with studies suggesting a surgical margin of 1-2 cm [7,8]. Patients receiving early treatment have a good prognosis, with survival rates exceeding 90% at five years in primary EMPD cases that do not invade beyond the epidermis [5,6,8].

## Conclusions

EMPD in men is a rare and challenging condition due to its clinical and histopathological similarity to other cutaneous pathologies, frequently leading to delayed diagnoses. This case highlights the importance of an interdisciplinary approach, including biopsy evaluation with immunohistochemical studies, for accurate diagnosis and the necessity of timely surgical interventions with negative margins to optimize prognosis. Close follow-up and imaging studies are essential to rule out associated malignancies and prevent complications. Early identification and appropriate management can significantly improve outcomes in these patients.
